# A specially designed domed‐cones template for needles (seeds) fixation and incline insertion in prostate implant brachytherapy

**DOI:** 10.1120/jacmp.v17i1.5833

**Published:** 2016-01-08

**Authors:** Zhao‐Sheng Yin, Shi‐Qiang Tang, Jun‐Wen Shi, Fen Chen, Zi‐Wei Li, Jia‐Ming Wu, Yee‐Min Jen

**Affiliations:** ^1^ Radiotherapy Center, Chenzhou No.1 People's Hospital Hunan China; ^2^ Department of Radiation Oncology Yee‐Ren Hospital Tao Yuan City Taiwan

**Keywords:** prostate needles (seeds) implant, domed‐cones template, parallel shift protractor, brachytherapy, presimulation

## Abstract

The construction of a conventional prostate needle (seeds) implant template restricts needles tilting or incline insertion when it is necessary to approach a seminal vesicle or to avoid the obstruction of symphysis pubis. To overcome the disadvantages of conventional templates, we developed a special template for guiding needles incline insertion and fixation for prostate needle implant. Phantom needles implantation was performed. Two acrylic boards, each 7.5 cm in width by 7.5 cm in length and 0.5 cm thickness, were drilled with a set of domed holes and cones with embedded template ball inside this combination to provide firm grip and fixation in prostate needle implantation. The specially designed domed‐cones combination acrylic board provides a needle of up to 60° rotation flexibility application. Some areas that could not be covered in a conventional parallel needle holes template could now be covered by using this new template. The covering index of prostate radiation dosage is up to 84.5%. The specially designed domed‐cones acrylic board combination provides not only a reliable means of needle fixation and rotational function, but also a superior dose distribution in the anterior portion of the prostate and good coverage of a seminal vesicle. This special template is a feasible design for prostate needles or seeds implant brachytherapy.

PACS number(s): 87.57.uq

## I. INTRODUCTION

Prostate cancer is mainly treated by radiotherapy, which includes external beam radiation therapy (EBRT) and interstitial brachytherapy (IBT).^1^, ^2^, ^3^, ^4^ There are two types of IBT for prostate cancer — high‐dose‐rate needle implants, and permanent seeds implant.^5^, ^6^, ^7^, ^8^ High‐dose‐rate needle implant brachytherapy is most commonly used to treat prostate cancer in many institutions; it may sometimes be referred to as “needle implantation” or pin‐insertion surgery. The needles are inserted or implanted directly into, or very close to, the tumor. They deliver high doses of radiation to the tumor without affecting the normal healthy tissues around it. This means that the procedure is less damaging to surrounding normal organs than conventional radiation therapy, where the radiation is delivered from outside the body and must pass through other tissues before reaching the tumor. IBT can also be used as a boost treatment after EBRT, depending on the stage of the disease in such patients.

When needle implantation is carried out nowadays, an ultrasound probe is inserted into the rectum, and images from this probe are used to assess the size and shape of the prostate gland. This is used by the doctor to identify how to place the needles at the right position for each patient. Then, the needles are inserted in the exact locations identified.^9^, ^10^, ^11^, ^12^, ^13^

The needles are put into the target positions and between 15 and 20 needles are placed into the prostate. The doctor uses ultrasound and X‐ray images to make sure the needles are in the right place. A special computer software program is then used to make sure the prostate gland is completely covered by the right dose of radiation to ensure that all cancer cells present in the prostate have been satisfactorily treated. The seminal vesicles lie superior to the prostate under the base of the bladder and are approximately 6 cm in length. IBT consists of placement of multiple needles in the prostate through the perineum. The gland is located posterior to the pubic symphysis, superior to the perineal membrane, inferior to the bladder, and anterior to the rectum. With the patient in either head down or buttocks down (lithotomy position), the radiologists is not able to incline needle to avoid pelvic arch or rectum to reach the prostate.

**Figure 1:**
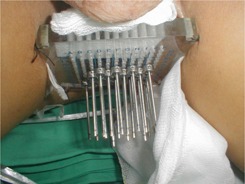
The conventional acrylic board with parallel holes template in prostate needle implant has disadvantages to tile or rotate when needles need to approach a seminal vesicle or to avoid the obstruction of symphysis pubis.

In general, the conventional acrylic board with parallel holes template in a prostate needle implant has the disadvantage of tiling or rotating when needles need to approach seminal vesicle or need to avoid the obstruction of symphysis pubis (Fig. 1).

Various types of transperineal templates are used to guide the implantation of needles and to secure them.^14^, ^15^, ^16^ Syed's et al.^(17)^ and Martinez's et al.^(18)^ multiple‐site universal perineal interstitial templates (MUPITs) are used mainly for better fixation on gynecologic interstitial treatment, but these grids or templates are unable to pinpoint the exact positions of the needles in the prostate since the needles cannot be inclined to avoid the pelvic arch or rectum to reach the prostate.^(19)^

In order to overcome the disadvantages of conventional templates and to solve the needles fixation and inclination problem to avoid needle withdrawal and bone obstruction in prostate needle implantation, we developed a special template for guiding needles insertion and fixation for prostate needle implants.

## II. MATERIALS AND METHODS

The initial design for needles fixation involves a dumbbell fixer. The dumbbell inner hollow is embedded in acrylic to avoid the needle slipping in and out and damaging soft tissue. The dumbbell can be rotated purposely like a screwdriver to fix or loosen the needles. Although this device can solve the fixation problem, the problem of needle inclination to avoid bone obstruction is still present. The development of a special template with fixation and inclination functions is described in the following sections.

### A. Domed‐cones template

**Figure 2:**
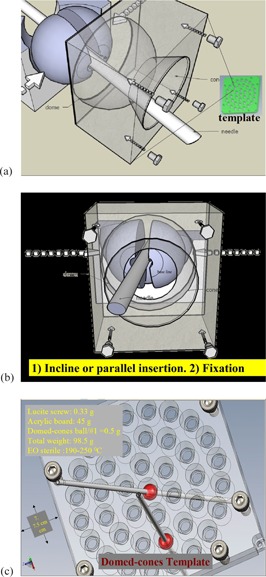
This special template (a) is designed to combine fixation and inclination functions in one template simultaneously. There is a base line (reference line) (b) on the template ball. When the base line is equidistant with the bottom of the volcanic crater, a parallel insertion of the needle is assured. This template (c) tolerates 190° C to 250° C high‐temperature sterilization. A total of 39 holes were concentrically arranged and the center of the circles aligned to the center of the prostatic urethra to avoid needle insertion into the urethra.

In order to combine fixation and inclination function in one needle template simultaneously, we designed a special template. This template consisted of two acrylic subplates, 7.5 cm in width by 7.5 cm in length, and 0.5 cm thick. There were 39 bowl holes sets in each acrylic plate, with a volcanic crater at the bottom of each bowl. The conical crater angle was 60° for needle inclination (Fig. 2(a)). When two subplate bowls bond together face to face, they form an empty headed ball shape for a template ball with needle hole. The needle's outer diameter is 1.21 mm, while the inner diameter of the tiny solid split ball hole is 1.20 mm. Therefore, when these two subplates are fixed by screws after needles insertion, two face‐to‐face bowls squeeze the inside solid template ball and hold the needle tightly in every hole. With our template, the needles are fixed and can be tilted with a great degree of inserted angle. The solid template ball is not actually hemisphere split cut, but with an asymmetric angle cutting to avoid the template ball needle hole drifted so that needle can be penetrated smoothly. There is a base line on the core ball. When the base line is equidistant with the bottom of the volcanic crater, the parallel insertion of the needle is ensured (Fig. 2(b)).

This template can tolerate 190° C to 250° C high temperature sterilization. A total of 39 holes were concentrically arranged. Urologist adjusts the center of the template aligned below the center of the prostatic urethra 0.5 cm by using a transrectum ultrasound probe to avoid needle insertion into the urethra (Fig. 2(c)).

### B. Template holder

**Figure 3:**
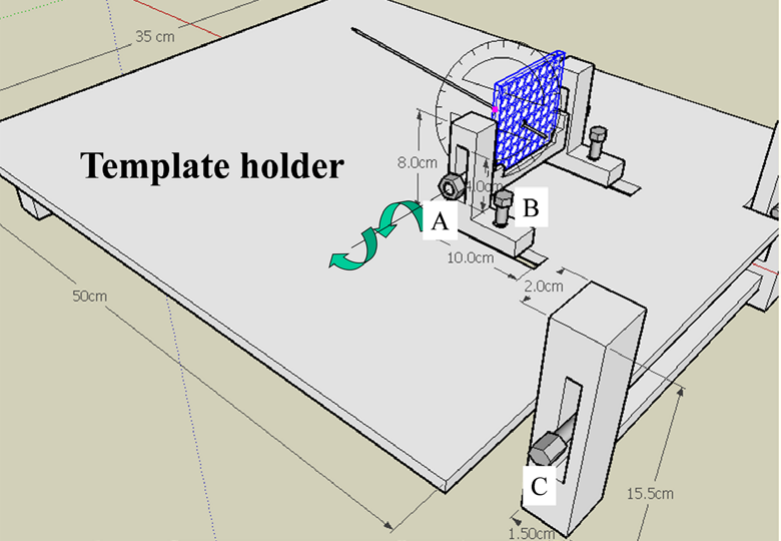
There are three twists, A, B, and C, in this figure. Twist A controls the pitch and up down movement, twist B controls back and forth movement, and twist C controls the up and down movement of the holder to fit the needle insertion requirement.

We designed a template holder for flexible needle adjustment and fixation (Fig. 3). There are three twists, A, B, and C, in this figure. Twist A controls the pitch and up down movement, twist B controls back and forth movement, and twist C controls the up and down movement of the holder to fit the needle insertion requirement.

### C. Manufacture of the prostate needle implantation phantom

**Figure 4:**
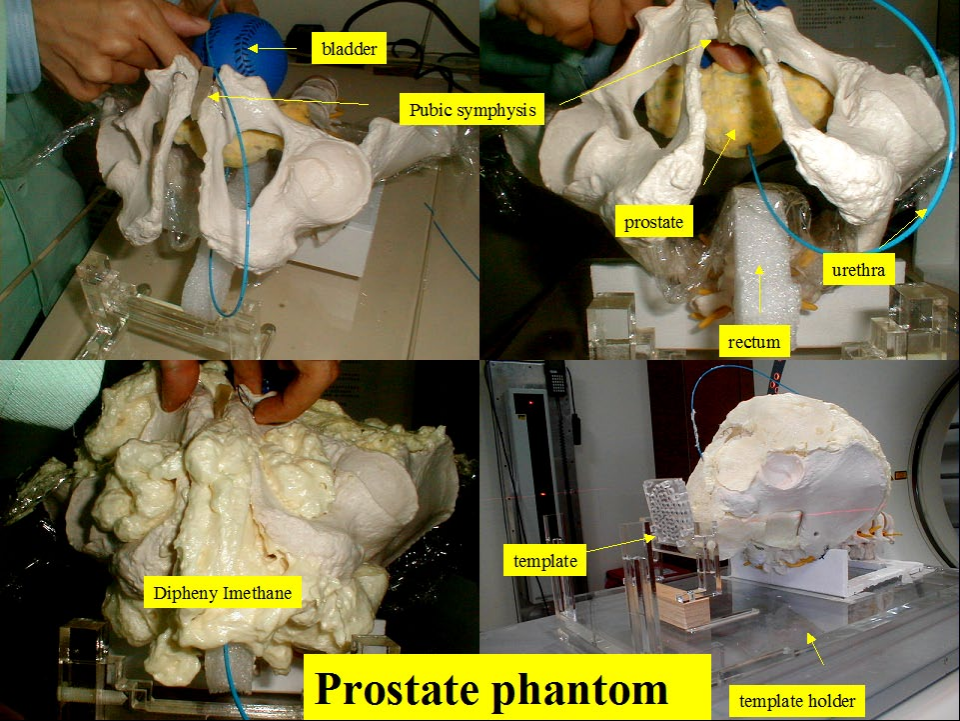
Manufacture of the prostate needle implantation phantom.

Figure 4 demonstrates the construction prostate of the implantation phantom. The bladder was substituted by a sponge; the prostate was made of strong sponge, and the urinary tract was made of rubber. All the organs were fixed by expanded polystyrene to form a phantom for needle implantation simulation.

### D. Needle implantation technique

This special template has so far been tested by phantom.

In order to perform needle implantation with this special template more efficiently, the following items were developed by using preplan system for needle insertion: 1) preplan before needle insertion, 2) needle implantation with the phantom, 3) parallel protractor.

#### D.1 Preplan before needle insertion

In this study, the optimal simulation system for prostate needle implantation is to pair up CT images with the special template and template holder. In this system, we preplan the needles setting on CT images to construct uniform dose distribution before patient treatment.

**Figure 5:**
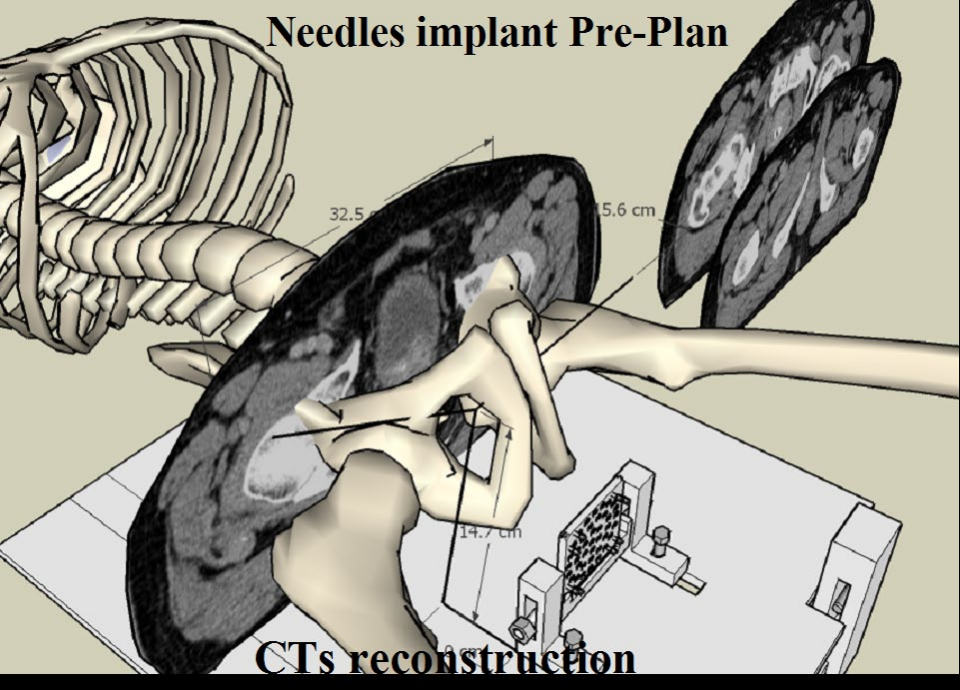
This procedure is to arrange CT images slice by slice reconstructed to a preplan system and to avoid needle puncture of the urethra. All needle insertions are conducted with equal geometry distribution between the needles to cover the prostate gland with incline insertion to avoid bone obstruction.

A planning CT scan of the prostate needle was done with a slice thickness of 2.5 mm. Urologist in real patient adjust the center of the template aligned below the center of the prostatic urethra 0.5 cm to avoid needle insertion into the urethra. The simulation procedure starts with CT image reconstruction and projection of the 39 concentric holes onto CT images to equal distributed on the lesion. Then, simulated needle insertion is conducted with equal distance between the needles to cover the prostate gland with inclination to avoid bone obstruction (Fig. 5). Brachytherapy planning was performed on PLATO planning system version 14.1 (Nucletron, Veenandaal, The Netherlands), whereby phantom prostate and organs at risk (OAR) were delineated.

#### D.2 Needle implantation with the phantom

The phantom was put on the template holder and then sent for CT scanning followed by simulation planning. The principle of simulation implantation is to arrange the needles ensuring they are well‐distributed in the prostate, while preventing damage to the surrounding organs such as the rectum and the urethra. In addition, inclination is used to avoid bone obstruction when performing needle insertion. The needle number and length outside the template must be recorded so that these simulation parameters can be applied to real patients.

#### D.3 Parallel protractor

**Figure 6:**
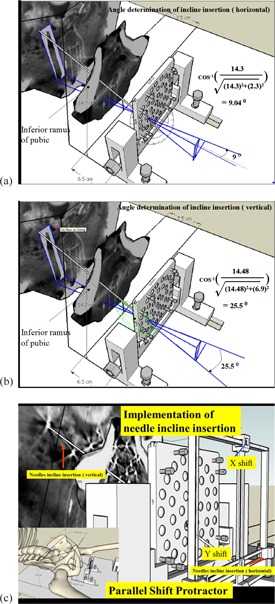
A needle angle that is parallel (a) or perpendicular (b) to the horizontal line is not able to be measured by such a small protractor. We designed a parallel protractor (c) to overcome this problem. With this device, the coordinate of the entry hole could be determined by moving the X‐Y shift. The depth of each simulated needle insertion is shown by the length outside the template. The identified needle number and its length outside the template are then used for real patient needles insertion.

The needle was inserted straight or inclined. If the needle was inserted inclined, the needle angle had to be determined for the inclined insertion (Fig. 6(a) and (b)). However, there is no such protractor available. Therefore, we designed a parallel protractor to overcome this problem (Fig. 6(c)). With this device, the coordinate of the entry hole could be determined by moving the X‐Y shift. The depth of each simulated needle insertion result the length outside the template. The identify needle number and its length outside the template are then used for real patient needles insertion.

### E. Reproduction of the simulation in real patients

The parameters obtained in previous simulation, such as template holder parameters and parallel protractor X and Y coordinator for each needle from the phantom, are shown in Fig. 6(c). The depth of each simulated needle insertion result the length outside the template. The identify needle number and its length outside the template are then used for real patient needles insertion.

### F. Verification of needle implantation

**Figure 7:**
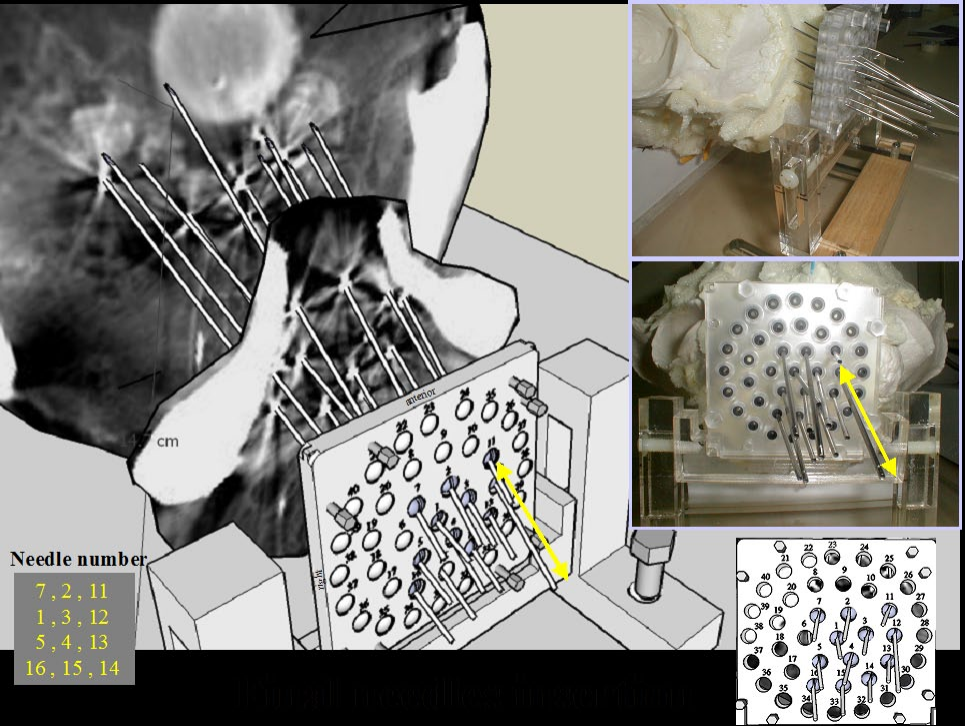
Phantom check of needle implantation according to preplan simulation.

We used a 16 cm needle for prostate needle implantation. The test implantation of each needle in the phantom was performed using the parameters obtained from the preplan (Fig. 7). This phantom test avoids unwanted needle insertion in real patients, such as penetration into the internal pudendal artery or the urinary tract.

### G. Patient procedures

#### G.1 Patient reference marker for needle insertion simulation

**Figure 8:**
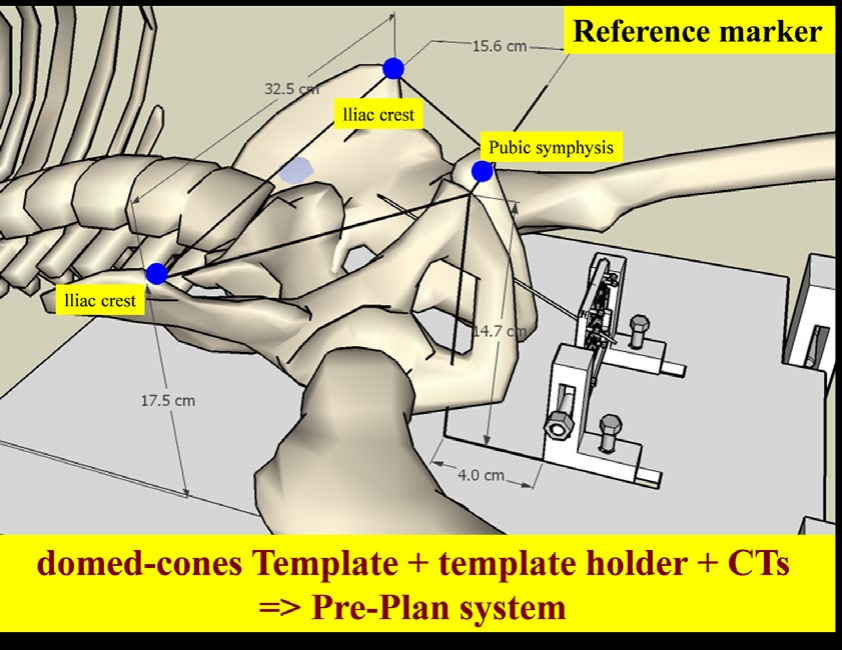
With patient bone marker (the distance from pubic symphysis to plate holder and the distance form right–left iliac crest to plate), the needles can be inserted and simulated with the aid of these markers.

This system integrates CT images and template holder to simulate the optimal patient‐template holder position for needle insertion. With bone marker of the distance from pubic symphysis to plate holder and the distance form right–left iliac crest to plate, the needles can be inserted with the aid of simulation in advance (Fig. 8).

#### G.2 Clinical practice on patient

The implant was performed using home‐made domed‐cones template under the guidance of TRUS imaging (Falcon 2101, BK Medicals, Herlev, Denmark). After administering spinal anesthesia, the patient was placed in the lithotomy position. A Foley's trilumen urinary catheter was inserted. After inserting a condom over the TRUS probe, it was introduced into the rectum and the bladder was identified first by visualizing the Foley's bulb so that orientation of other pelvic structures could be made.

The plate was positioned against the perineal wall and stitched to the skin. The stainless steel needles (with blind ends), 16 cm in length and with 1.21 mm inner diameter, were inserted one by one through the perineum. The first needle should be inserted into the prostate gland above the rectum anterior wall to avoid rectum damage, and then upper right or upper left of the gland, and from the anterior to the posterior aspect since the needles closer to the TRUS probe may obscure the anterior needle images. The needle movement was tracked to a desired depth by visualizing the tip of the needle seen by moving the TRUS probe in a forward direction. Any needle which caused accidental entry into the bladder, the rectum or the bowel was immediately withdrawn. After inserting the desired number of needles for adequate coverage of the prostate target area, the TRUS probe was taken out from the rectum and digital rectal examination was performed to rule out any needle penetration into the rectum. The needles were fixed with the help of screws tightened on an acrylic board to stress the template ball to prevent needle movement. Brachytherapy planning was performed on PLATO planning system version 14.1 (Nucletron), whereby prostate and organs at risk (OAR) were delineated. The contour drawn by the line joining the outermost needles on the CT slice constituted the boundary of the prostate. However, the craniocaudal extent of the target was determined by selecting the length of needles, keeping in mind the clinical and radiological findings. After finalizing the target and OAR volumes, implant needles were also marked on each slice in order to reconstruct the needle length. Using step size of 2.5 mm, a plan was generated. The dose was not prescribed to a particular point; rather, it was prescribed at the periphery of the prostate volume. Only dwell positions within the target volume were activated. After the plan approval, the patient was taken to the brachytherapy room (Microselectron HDR remote after‐loading unit) for treatment. The implant needles were removed immediately after completion of the treatment.

## III. RESULTS

Our specially designed domed‐cones template is positioned and sutured into the perineum. The transrectal ultrasound is positioned so that it can visualize the tumor and normal surrounding structures in both transverse and longitudinal planes. Needles are inclined or parallel inserted with this domed‐cones template into the target area under direct visualization through transverse imaging. The bladder and rectum can be directly imaged and thus avoided. Longitudinal imaging is then used to guide the needles to the appropriate depth. In addition, this template can be used to avoid needles slipping in and out and damaging soft tissue or maltreating the target.

### A. Radiation dose distribution

**Figure 9:**
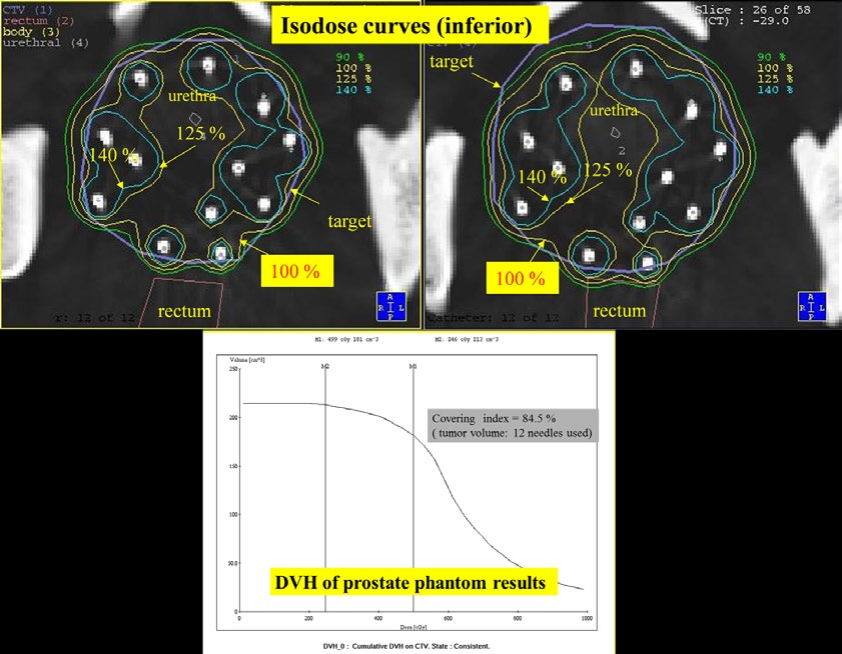
The final dose distribution (isodose curves) is presented. Usually, the urethra is restricted to under 125% of the prostate dose. The covering index is 84.5% (covering index is defined as M1 volume / M2 volume, and the prescribed dose is 500 cGy in this demonstration).

The final dose distribution (isodose curves) is presented in Fig. 9. The optimal radiation dose distribution is formed by adjusting Ir‐192 source position and dwelling time. Usually, the urethra is restricted to under 125% of the prostate dose. Tumor covering index is defined as M1 volume/M2 volume. M1 volume means the total volume encompassed by the prescribed dose; the covering index is 84.5% (the prescribed dose is 500 cGy in this demonstration).

## IV. DISCUSSION

IBT is generally reserved for patients either with extensive pelvic and/ vaginal or prostatic disease in an attempt to improve local control or with anatomy not allowing interstitial brachytherapy with standard applicators. The aim of this technique is to tailor the dose of irradiation to the prostate of the patient with a better target volume coverage. Originally, prostate needle implants were performed with free‐hand placement of the needle template. The development of multiple‐site universal perineal interstitial template (MUPIT) by Martinez and the Syed‐Neblett template resulted in a better needle positioning. Despite an improvement in the technological approach of these techniques, the issue of incline insertion and fixation in prostate needle implant has not been thoroughly solved. This special domed‐cones–shaped template is aimed to decrease the risk of complications the conventional template nowadays used.

Two main perineal templates types have been described in the literature: the multiple‐site universal perineal interstitial template (MUPIT) and the Syed‐Neblett template.

The MUPIT was designed to treat not only gynecological malignancies but also prostatic, anorectal and perineal tumors. This applicator consists of two acrylic cylinders, one acrylic template, and a cover plate.

The template has arrays of holes used as guides for the needles. Guide holes are designed to allow the inserted trocars lie in parallel horizontal planes, perpendicular to the template plane, insuring an adequate geometry of the application. The planes are spaced vertically 1 cm apart. The MUPIT template was mainly designed for GYN cases. The vaginal cylinder is placed in the vagina with a fixation to the uterine catheter. The template is then fixed to the cylinder with sutures to the perineum. The needles are inserted to the appropriate depth, starting with the needles near the rectum, with one finger inside the rectum to avoid rectal perforation while an assistant pulls gently on the sutures. A second cylinder is placed in the rectum and sutured to the template to assure a fixed distance between the vagina and the rectum and to push away the posterior rectal wall. The rest of the needles are inserted around the vagina and the sutures initially stitched through the tumor and the normal tissues are removed. The template is sutured to the perineum and the cover plate is placed over the template to prevent the needles from displacement. When MULPIT template is used for prostatic cancer needle implant, the needles can only inserted parallel to vaginal/rectum cylinder, while the domed‐cones template can perform incline insertion and fixation.

The Syed‐Neblett template, originally described as the “transperineal parametrial butterfly,” is based upon the same principle. It consists of two superimposed plastic plates, each 1.2 cm thick, held together by screws. Predrilled holes are designed in a concentric “butterfly” pattern to accept the guide needles. Some needles are designed on the surface of the applicator and used in case of anatomical distortions not allowing a proper placement of conventional intracavitary applicators. The needles are fixed to the template by tightening the screws between the two plastic plates. A central hole in the template allows a plastic vaginal cylinder with a central opening which accepts the conventional uterine catheter. Again, when Syed‐Neblett template is used for prostatic cancer needle implant, the needles can only inserted parallel to the plastic vaginal cylinder, while the domed‐cones template can perform incline insertion and fixation.

## V. CONCLUSIONS

The combination of a special template and parallel protractor with CT images form a “prostate needle (seeds) implantation simulation system” provides a practical procedure for a radiation oncologist and medical physicist in prostate needle (seeds) implantation. In order to perform needle implantation with this special template more accuracy, the preplan system for needle insertion is applied. These procedures slow down the clinical practice. But it can be resolved by using the real‐time treatment planning system combined the transrectal ultrasound image. The highly efficient procedure by applying the real‐time treatment planning system and the benefit of the incline insertion with needles fixation of the domed‐cone shape template is incomparable.

## ACKNOWLEDGMENTS

This study (N 2014‐016) was sponsored by Radiotherapy Center, Chenzhou No.1 People's Hospital, Hunan, China, and E‐Da hospital EDAHT99001, and the National Natural Science Foundation of China (Grant No. 81402291, to J‐W Shi). The authors declare no conflicts of interest.

## Supporting information

Supplementary Material

Supplementary Material

Supplementary Material

Supplementary Material
